# Human sensory-like neuron surfaceome analysis

**DOI:** 10.1371/journal.pone.0320056

**Published:** 2025-04-02

**Authors:** Maximilian Breyer, Stephanie Lamer, Andreas Schlosser, Nurcan Üçeyler

**Affiliations:** 1 Department of Neurology, University Hospital Würzburg, Würzburg, Germany; 2 Rudolf Virchow Center, Center for Integrative and Translational Bioimaging, University of Würzburg, Würzburg, Germany; 3 Würzburg Fabry Center for Interdisciplinary Therapy (FAZIT), University Hospital Würzburg, Würzburg, Germany; Indiana University School of Medicine, UNITED STATES OF AMERICA

## Abstract

Acral and triggerable pain is a hallmark of diseases involving small nerve fiber impairment, yet the underlying cellular mechanisms remain elusive. A key role is attributed to pain-related proteins located within the neuronal plasma membrane of nociceptive neurons. To explore this, we employed human induced pluripotent stem cell-derived sensory-like neurons and enriched their surface proteins by biotinylation. Samples from three independent cell differentiations were analyzed via liquid chromatography tandem mass spectrometry. Detected proteins were categorized by cellular location and function, followed by generating an interaction network for deregulated surface proteins. Gene expression of selected proteins was quantified using real-time PCR. A comparative analysis was performed between a patient with Fabry disease (FD) and a healthy control, which we used as model system. We successfully extracted surfaceome proteins from human sensory-like neurons, revealing deregulation of 48 surface proteins in FD-derived neurons. Among the candidates with potential involvement in pain pathophysiology were CACNA2D3, GPM6A, EGFR, and ABCA7. Despite the lack of gene expression differences in these candidates, the interaction network indicated compromised neuronal network integrity. Our approach successfully enabled the extraction and comprehensive analysis of the surfaceome from human sensory-like neurons, establishing a novel methodological framework for investigating human sensory-like neuron biology and cellular disease mechanisms.

## Introduction

Acral neuropathic pain accompanied by par- or dysesthesias is a hallmark of diseases involving small nerve fiber impairment [1]. Although the cellular mechanisms remain largely unclear, studies using human-derived cell culture systems have gained significant relevance [2]. The analysis of neuronal protein expression is essential, with particular focus on alterations in the expression and functionality of pain-related ion channels, such as voltage-gated sodium and potassium channels. In this context, the advent of induced pluripotent stem cell (iPSC)-based *in vitro* systems provides an opportunity to study patient-derived sensory-like neurons. Coupled with mass spectrometry, this technique has allowed to characterize the proteome of human neurons and assess the proteomic impact of neurotoxic conditions [3–5]. However, the surfaceome, which encompasses proteins residing in the plasma membrane with extracellular domains, remains underrepresented in these datasets due to its low abundance and challenging extraction [6]. Biotin-based chemical labeling has previously been employed to successfully investigate cortical neuron surfaceomes of rodents [7] utilizing an efficient strategy to enrich surface proteins [6].

To apply iPSC-based methodologies, a genetic background is crucial to link findings to specific gene variants. Fabry disease (FD, OMIM 301500) is an X-linked multisystem disorder associated with acral neuropathic pain [8,9] based on small fiber neuropathy [10]. FD is caused by mutations in the α-galactosidase A (*GLA*) gene, leading to deficient lysosomal GLA enzyme activity [11], which results in the accumulation of the glycosphingolipid globotriaosylceramide (Gb3) in lysosomes [12]. Previous studies have demonstrated Gb3 accumulation in plasma membranes and deregulation of membrane organization in FD-affected cell types [13–17]. Animal models suggest a direct impact of Gb3 on the expression and function of pain-related ion channels, including Nav1.7 and TRPA1, within nociceptive neuron plasma membranes [18,19]. However, proteomic data from human FD cells remain scarce, with most studies focusing on systemic proteomes, such as plasma or urine [20–22]. In this study, we aimed to characterize the surfaceome of iPSC-derived sensory-like neurons and used sensory-like neurons generated from a FD patient as a model.

## Materials and methods

### Source and cultivation of iPSC

iPSC derived from a FD patient carrying the pathogenic nonsense variant p.Q357X (c.1069C > T) and a healthy control person without genetic variations in *GLA,* previously shown to differ significantly with absent α-GAL A activity and emerging Gb3 accumulations in FD iPSC [2], were employed in this study. Study subjects had been recruited at the Fabry Center for Interdisciplinary Therapy (FAZIT) University Hospital Würzburg between 1.7.2015 and 31.12.2021. Data were accessed for research purposes 1.7.2020 until 30.6.2023. iPSC were cultured in StemMACS™ iPS-Brew XF, human medium (Miltenyi Biotec, Bergisch Gladbach, Germany) supplemented with 100 U/ml penicillin/streptomycin (Pen/Strep, Thermo Fisher Scientific, Waltham, MA, USA). Cells were grown on hESC qualified Matrigel- (Corning, Corning, NY, USA) coated 6-well plates (Greiner Bio-One, Kremsmünster, Austria) under 5% CO_2_ and 37°C. Medium was changed daily and cells were passaged twice a week at a confluence of 80% using 2 mM EDTA (Thermo Fisher Scientific, Waltham, MA, USA) in phosphate buffered saline (PBS, Sigma-Aldrich, St. Louis, MS, USA). For the first 24 hours after passaging, medium was supplemented with 10 µ M Y27632 (Miltenyi Biotec, Bergisch Gladbach, Germany) to suppress apoptosis. The study was approved by the Ethics Committee of the University of Würzburg Medical Faculty (#26/19). Informed Consent Statement: Informed written consent was obtained from all subjects involved in the study before inclusion. All procedures performed in this study were carried out in accordance with German and international law, and the Declaration of Helsinki.

### Sensory-like neuron differentiation

In a ten-day protocol, iPSC were differentiated to sensory-like neurons [2]. In brief, differentiation medium was changed daily with altered composition of small molecules every other day. To trigger neuralization, iPSC were exposed to medium containing the dual SMAD inhibitors LDN-193189 (Stemcell Technologies, Vancouver, Canada) and SB431542 (Miltenyi Biotec, Bergisch Gladbach, Germany) for two days. From day three on, generation of nociceptive sensory-like neurons was induced by additionally supplementing medium with SU5402, DAPT (both Sigma-Aldrich, St. Louis, MO, USA), and CHIR99021 (Axon Medchem, Groningen, Netherlands) small molecules to modulate notch-receptor, FGF-receptor, and WINT-signaling. Simultaneously, concentration of B-27™ Plus and N-2 supplement (both Gibco, Waltham, MA, USA) was increased stepwise to enhance neuronal growth and survival. At day ten, differentiated neurons were detached with TrypLE™ Express (Gibco, Waltham, MA, USA) and seeded on growth factor reduced Matrigel- (Corning, Corning, NY, USA) coated 6-well plates. Neurons were matured in medium containing β-nerve growth factor (β-NGF), brain-derived neurotrophic factor (BDNF), and glial cell-derived neurotrophic factor (GDNF, all Peprotech, Rocky Hill, NJ, USA) for six weeks. Bright-field images of mature neurons were acquired with a Leica DM IL LED inverse microscope equipped with a DMC2900 camera (both Leica, Wetzlar, Germany).

### Surface protein extraction

Plasma membrane proteins were extracted from neuronal cultures after six weeks of maturation with the Pierce™ Cell Surface Protein Biotinylation and Isolation Kit (Thermo Fisher Scientific, Waltham, MA, USA). For both FD and healthy control neurons, three independent cell differentiations were prepared and analyzed in independent paired proteomic runs (three replicate experiments). For each differentiation, neurons from two complete 6-well plates were pooled. In brief, cells were washed with PBS and covered with sulfo-NHS-SS-biotin solution for ten minutes at room temperature. Cells were then washed twice with ice-cold tris-buffered saline (TBS), scraped into fresh TBS, and centrifuged at 4°C. Lysis buffer was supplemented with complete protease inhibitor (EDTA-free, Roche, Basel, Switzerland) and cells were lysed for 30 minutes on ice. After centrifugation, clarified supernatant was extracted and biotin-labeled proteins were loaded to a NeutrAvidin-agarose column for 30 minutes. During incubation, columns were kept on a microtube shaker (bioSan, Riga, Latvia) and inverted 20 times every five minutes. After washing steps, columns were incubated similarly for 30 minutes with elution buffer containing 10 mM dithiothreitol. After centrifugation, the eluate was kept in low-protein-binding tubes (Eppendorf, Hamburg, Germany) on dry ice until gel electrophoresis. For Matrigel-only control, two complete 6-well plates were coated with growth factor-reduced Matrigel, incubated for six weeks under 5% CO_2_ and 37°C, and treated with the same protocol. For the first out of three paired experiments, additional samples derived from cells that were not treated with biotin prior to NeutrAvidin pulldown were included to assess the specificity of the enrichment column. An exemplary image of the resulting gel lane (see next paragraph on Gel electrophoresis) compared to the biotin-treated samples is provided in S1 Fig, showing that no relevant protein quantities were detected in the untreated samples.

### Gel electrophoresis

Protein was precipitated overnight at -20°C with fourfold volume of acetone. Pellets were washed three times with acetone at -20°C. Precipitated proteins were dissolved in NuPAGE® LDS sample buffer (Thermo Fisher Scientific, Waltham, MA, USA), reduced with 50 mM DTT at 70°C for ten minutes, and alkylated with 120 mM Iodoacetamide at room temperature for 20 minutes. Separation was performed on NuPAGE® Novex® 4-12% Bis-Tris gels (Thermo Fisher Scientific, Waltham, MA, USA) with MOPS buffer according to manufacturer’s instructions. Gels were washed three times for five minutes with water and stained for 60 minutes with Simply Blue™ Safe Stain (Thermo Fisher Scientific, Waltham, MA, USA). After washing with water for one hour, each gel lane was cut into 15 slices.

### In-gel digestion

The excised gel bands were destained with 30% acetonitrile in 0.1 M NH_4_HCO_3_ (pH 8), shrunk with 100% acetonitrile, and dried in a vacuum concentrator (Concentrator 5301, Eppendorf SE, Hamburg, Germany). Gels were digested with 0.1 µg trypsin per gel band overnight at 37°C in 0.1 M NH_4_HCO_3_ (pH 8). After removing the supernatant, peptides were extracted from the gel slices with 5% formic acid, and extracted peptides were pooled with the supernatant.

### Liquid chromatography tandem mass spectrometry (NanoLC-MS/MS) analysis

NanoLC-MS/MS analysis was performed on an Orbitrap Fusion (Thermo Fisher Scientific, Waltham, MA, USA) equipped with a PicoView Ion Source (New Objective, Littleton, MA, USA) and coupled to an EASY-nLC 1000 liquid chromatograph (Thermo Fisher Scientific, Waltham, MA, USA). Peptides were loaded on a trapping column (2 cm x 150 µm ID, PepSep, Marslev, Denmark) and separated on a capillary column (30 cm x 150 µm ID, PepSep), both packed with 1.9 µm C18 ReproSil (Dr. Maisch HPLC GmbH, Germany) and separated with a 30-minute linear gradient from 3% to 30% acetonitrile and 0.1% formic acid and a flow rate of 500 nl/minute. Mass spectrometry (MS) and MS/MS scans were acquired in the Orbitrap analyzer with a resolution of 60,000 for MS scans and 30,000 for MS/MS scans. Higher energy collisional dissociation (HCD) fragmentation with 35% normalized collision energy was applied. A Top Speed data-dependent MS/MS method with a fixed cycle time of three seconds was used. Dynamic exclusion was applied with a repeat count of one and an exclusion duration of 30 seconds; singly charged precursors were excluded from selection. Minimum signal threshold for precursor selection was set to 50,000. Predictive automatic gain control (AGC) was used with an AGC target value of 4x10^5^ for MS scans and 5x10^4^ for MS/MS scans. EASY-IC ion source was used for internal calibration.

### MS data analysis

Raw MS data files were analyzed with MaxQuant version 1.6.2.2 [23]. Database was searched with Andromeda, which is integrated in the utilized version of MaxQuant. The search was performed against the UniProt Human Reference Proteome database (June 2022, UP000005640, 79684 entries). Additionally, the contaminants database integrated in MaxQuant was used. The search was performed with tryptic cleavage specificity and three allowed miscleavages. Protein identification was under control of the false-discovery rate (FDR; < 1% FDR on protein and peptide spectrum match [PSM] level). In addition to MaxQuant default settings, the search was performed against following variable modifications: Protein N-terminal acetylation, Gln to pyro-Glu formation (N-term. Gln) and oxidation (Met). Carbamidomethyl (Cys) was set as fixed modification. Further data analysis was performed using R scripts developed in-house. Label-free quantitation (LFQ) intensities were used for protein quantitation. Proteins with less than two razor/unique peptides were removed. Missing LFQ intensities were imputed with values close to baseline. Data imputation was performed with values from a standard normal distribution with a mean of the 5% quantile of the combined log10-transformed LFQ intensities and a standard deviation of 0.1. To identify deregulated proteins, mean log2-transformed protein ratios of FD versus Ctrl samples were derived from the three replicate experiments. In addition, the R package limma was used to calculate Benjamini-Hochberg adjusted p-values and FDR [24]. Proteins with an adjusted p-value < 0.05 were considered deregulated.

### Enrichment analysis

For enrichment analysis in detected and deregulated proteins, respectively, gene ontology (GO) annotation GO_CC_DIRECT was performed with the Database for Annotation, Visualization and Integrated Discovery (DAVID) [25–27]. In addition, functional annotation clustering was performed with DAVID. Proteins were tested against the DAVID default set of categories including UniProt [28,29] keyword, gene ontology (GO) term, and Kyoto Encyclopedia of Genes and Genomes (KEGG) pathway databases. Annotated categories were clustered using default settings, and p-value of enrichment was corrected with the method of Benjamini and Hochberg [30]. In addition, cellular and subcellular expression as well as function of selected proteins was manually assessed using GeneCards [31,32], Human Protein Atlas (HPA) [33,34], GO, and UniProt databases.

### Gene expression analysis

FD and healthy control neurons were lysed with Quiazol after six weeks of maturation, respectively, and total RNA was isolated using the miRNeasy Micro Kit (both Quiagen, Milden, Germany). RNA concentration and quality were determined with a NanoDrop One spectrophotometer (Thermo Fisher Scientific, Waltham, MA, USA), and 250 ng RNA were used for cDNA synthesis via TaqMan Reverse Transcription Reagents (Thermo Fisher Scientific, Waltham, MA, USA). Quantitative real-time polymerase chain reactions (qPCR) were performed in duplex for target genes and an endogenous control (*GAPDH*) using TaqMan^®^ Gene Expression Assays (Thermo Fisher Scientific, Waltham, MA, USA; S1 Table) with 8.75 ng cDNA per well on a Quantstudio 3 qPCR machine (Thermo Fisher Scientific, Waltham, MA, USA). Expression was determined with the ΔΔC_t_ method and data from four differentiations, prepared separately from the differentiations used for proteomic analysis, were acquired for FD and healthy control sensory-like neurons, respectively. Statistical analysis was performed on SPSS software version 27 (IBM, Ehningen, Germany) using the Mann-Whitney-U test. Differences were considered significant at p < 0.05 and graphs were generated in Graphpad PRISM software version 9.3.1 (GraphPad Software, Inc., La Jolla, CA, USA).

### Protein-protein interaction

For identification of interaction clusters and proteins of strong connectivity, the Search Tool for the Retrieval of Interacting Genes (STRING) was used to generate a protein-protein interaction (PPI) network with a medium required interaction score of 0.4 [35,36]. Biological function of mapped proteins was individually assessed using GeneCards, UniProt, and the HPA.

## Results

### Successful enrichment of sensory-like neuron cell surface proteins

**Fig 1:**
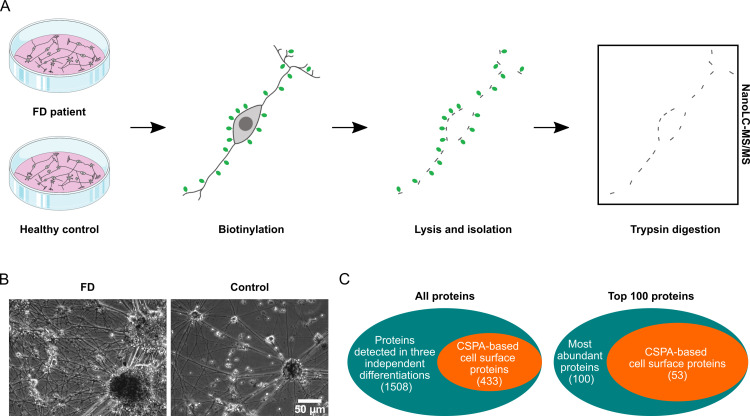
Concept and result of surface protein enrichment in iPSC-derived. Sensory-like neurons were processed as follows: (A) Matured sensory-like neurons were incubated with sulfo-NHS-SS-biotin solution in vitro. Biotin binds to extracellular portions of plasma membrane proteins, enabling the isolation of surface protein fractions through NeutrAvidin-mediated pulldown after cell lysis. Following this, biotin was cleaved by trypsin digestion, and surfaceome-enriched protein samples were subjected to mass spectrometry analysis. (B) Representative phase-contrast photomicrographs of FD and healthy control sensory-like neurons, taken after six weeks of maturation and before biotin labeling, show dense neuronal networks comprising both clusters and single cells for both genotypes. (C) An Euler diagram of CSPA-based surface protein annotation within the complete set of 1,508 detected proteins, as well as the top 100 most abundant proteins, highlights a distinct enrichment of cell surface proteins. Abbreviations: CSPA = Cell Surface Protein Atlas, FD = Fabry disease, iPSC = induced pluripotent stem cells, NanoLC-MS/MS = liquid chromatography-tandem mass spectrometry.

Via biotinylation followed by mass spectrometry (Fig 1A, 1B), we identified 1,508 distinct proteins in sensory-like neuron cultures derived from FD patients and controls, consistently observed across three independent experiments (S2 Table). Gene Ontology (GO) annotation using the GO_CC_DIRECT category revealed that 47% of these proteins are associated with the “plasma membrane.” Strikingly, within the top 100 most abundant proteins, this proportion increased to 67%. Functional annotation clustering of the top 100 proteins via DAVID further underscored the enrichment of membrane-associated proteins, with the most prominent cluster comprising the GO term “plasma membrane” alongside UniProt keywords “cell membrane” and “membrane” (enrichment score 8.68, S3 Table). Given that membrane proteins represent only 25–30% of all human protein-coding genes, our data highlights a significant overrepresentation of this class in the analyzed proteome [37,38]. Notably, other studies employing biotinylation- and mass spectrometry-based enrichment approaches report plasma membrane annotation in the range of 24-54% of all identified proteins [39–41]. Although these reports are based on other cell or tissue types, a plasma membrane annotation of 47%, as presented in our study, indicates a rather high level of enrichment within the range achievable with this approach.

For a protein to be classified within the surfaceome, it must contain an extracellular domain. Therefore, the identified plasma membrane proteins were subsequently cross-referenced with the validated surfaceome dataset from the Cell Surface Protein Atlas (CSPA) to ensure accurate classification [42,43]. We found that 29% of all detected proteins and 53% of the top 100 most abundant proteins matched the validated surfaceome dataset (Fig 1C). Considering that cell surface proteins are estimated to constitute approximately 10–20% of all human gene products, these results demonstrate a pronounced enrichment of surfaceome components within the neuron-derived proteome [44,45]. Notably, mass spectrometry analysis of Matrigel-only samples detected just six proteins with low peptide counts (S4 Table), of which two—lysozyme C (LYZ) and desmoplakin (DSP)—were also present in the cell culture-derived samples. This minimal overlap indicates that Matrigel had no significant impact on the overall protein composition of our dataset.

### Detection of proteins characterizing the neuronal cell culture

Consistent with the neuronal origin of the analyzed cell cultures, the top five most abundant proteins included neural cell adhesion molecule 1 (NCAM1), contactin 1 (CNTN1), and L1 cell adhesion molecule (L1CAM). Additionally, we detected pan-neuronal surface markers such as gamma-enolase (ENO2) and postsynaptic neuroligin-2 (NLGN2). Notably, distinct expression of pain-related ion channels was observed, including voltage-gated sodium channel Nav1.7 (SCN9A) and P2X purinoceptor 3 (P2RX3) [46,47], the latter being exclusive to nociceptors [48,49]. Moreover, the expression of tropomyosin receptor kinase B (NTRK2) and RET proto-oncogene (RET) indicates a predominant presence of non-peptidergic nociceptors, previously identified as the functionally affected neuronal subpopulation in FD neurons [50,51]. A conclusive statement on the non-peptidergic character of the investigated neurons can, however, not be made since expression of relevant peptidergic markers such as CGRP or substance P were not assessed in this study.

### Deregulated surface proteins

**Fig 2:**
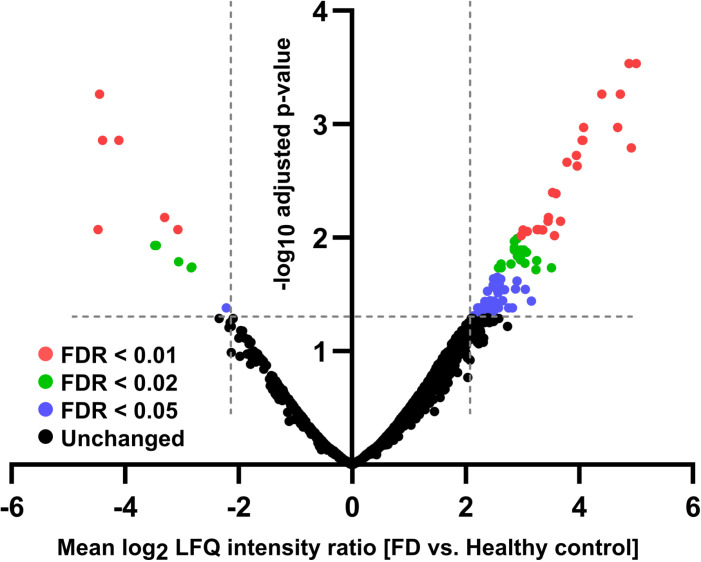
Volcano plot of altered surfaceome in FD sensory-like neurons normalized to a healthy control line. Samples from three independent differentiations, pairwise analyzed in independent proteomics experiments, were included for each group. Adjusted p-values were calculated using the R package “limma.” Dotted horizontal and vertical lines represent the p-value and LFQ intensity ratio thresholds, respectively. In FD sensory-like neurons, 86 proteins were upregulated, while 12 were downregulated compared to healthy controls. Abbreviations: FD =  Fabry disease, LFQ =  label-free quantitation.

We compared the surfaceome composition between FD and healthy control nociceptors, identifying 98 differentially regulated proteins in the FD neuronal cultures, 86 upregulated and 12 downregulated (Fig 2). To specifically isolate surface proteins, these deregulated proteins were cross-referenced with the CSPA and further assessed for extracellular and/or transmembrane domains using the UniProt database (S5 Table). Forty-eight proteins (49%) met at least one of these criteria and were classified as part of the surfaceome. Among the most deregulated proteins (p <  0.01), we identified several neuronal candidates with a potential link to nerve pathology such as: calcium voltage-gated channel auxiliary subunit alpha2delta 3 (CACNA2D3, p <  0.01) [52], neuronal membrane glycoprotein M6-A (GPM6A, p <  0.001) [53,54], ATP-binding cassette subfamily A member 7 (ABCA7) [55], and epidermal growth factor receptor (EGFR, p <  0.01) [56,57]. While the latter is more recognized to be expressed in other cell types including mesenchymal stem cells, also expression in neurons is increasingly reported [58], and even a potential role in pain signaling. However, further analysis of these candidates was not possible due to the small sample size.

### No evidence for a correlation between surfaceome deregulation and gene expression

**Fig 3:**
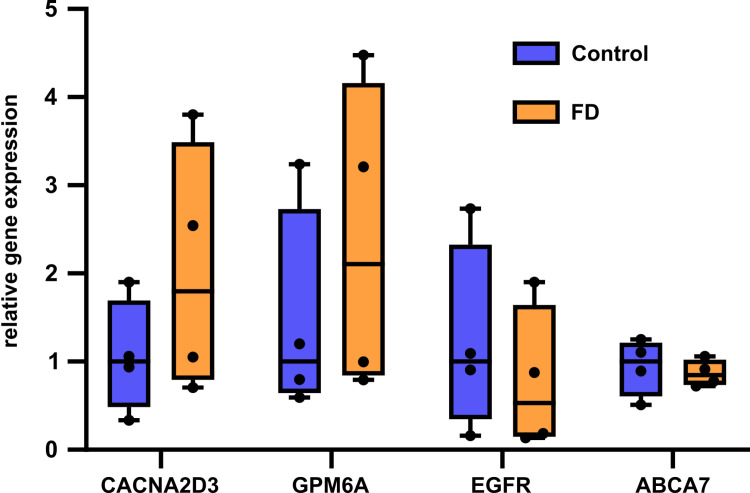
qPCR analysis of CACNA2D3, GPM6A, EGFR, and ABCA7 gene expression in iPSC-derived sensory-like neurons. All four targets with potential relevance to FD pathology exhibited expression at the mRNA level, with no significant differences found between FD and control neurons (nFD = 4, nControl = 4 independent differentiations). Data is shown as normalized fold change with regard to one of the control line values that was chosen as the calibrator for each gene assessed, respectively. Abbreviations: ABCA7 =  ATP-binding cassette subfamily A member 7; CACNA2D3 =  calcium voltage-gated channel auxiliary subunit alpha2delta 3; EGFR =  epidermal growth factor receptor; FD =  Fabry disease; GPM6A =  neuronal membrane glycoprotein M6-A; iPSC =  induced pluripotent stem cells; qPCR =  quantitative polymerase chain reaction.

To assess whether the observed differences in the neuronal plasma membrane were mirrored at the gene expression level, we conducted qPCR analysis for CACNA2D3, GPM6A, EGFR, and ABCA7, using samples from four separately prepared differentiations for FD and healthy control sensory-like neurons, respectively. While expression of these genes was confirmed by Ct values well below 32, no differences in mRNA levels were found between FD patient-derived and healthy control sensory-like neurons (Fig 3).

### Deregulated surface protein interactions suggest maladaptive network formation in FD neurons

**Fig 4:**
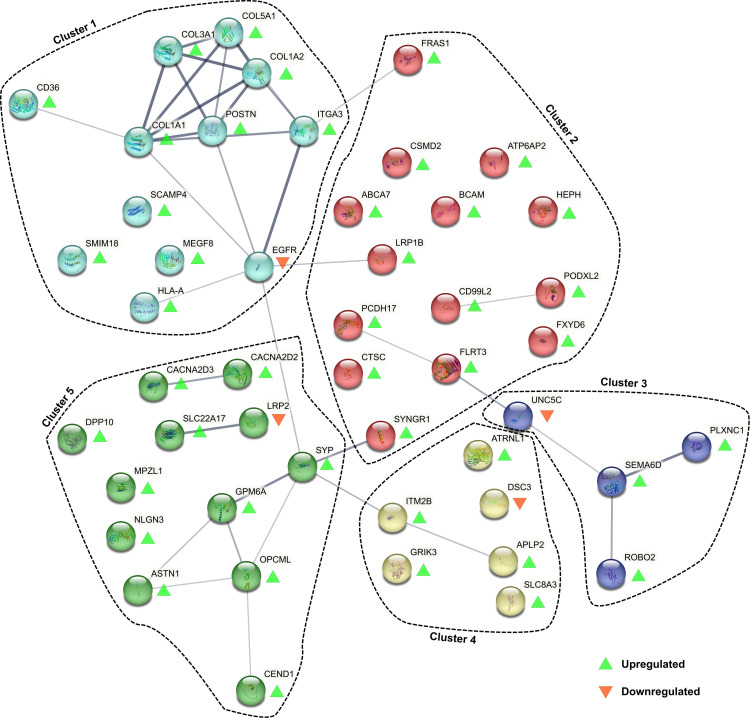
Protein-protein-interaction network generated from the deregulated surface proteins with STRING database. The network revealed 38 connections, exceeding the expected number of 10 (average interaction degree: 1.58). K-means clustering was conducted with a pre-defined cluster number of five. A detailed description of these interactions is provided in S6 Table. Abbreviations: FD =  Fabry disease, STRING =  Search Tool for the Retrieval of Interacting Genes.

To investigate interactions among the 48 deregulated surface proteins, STRING analysis was performed, revealing 38 interactions and identifying five distinct protein clusters through k-means clustering (Fig 4). Cluster 1 was predominantly composed of extracellular matrix components, including COL5A1, COL3A1, COL1A2, COL1A1, and POSTN. Cluster 3 contained a subnetwork of transmembrane receptors, such as ROBO2, PLXN1, and its ligand SEMA6D, as well as UNC5C—all involved in axon guidance and neuronal development, suggesting altered axon branching and/or innervation issues in the investigated FD neurons. Cluster 5 included GPM6A, synaptophysin (SYP), ASTN1, and CEND1, proteins linked to neuronal plasticity, adhesion, and differentiation, further pointing to potential disruptions in neuronal network integrity.

## Discussion

In this study, we present a protocol for the enrichment of cell surface proteins from human iPSC-derived sensory-like neurons, followed by their expression analysis using mass spectrometry. This approach offers a novel framework, particularly for the targeted investigation of membrane ion channel proteins in excitable human cells, providing valuable insights into disease pathophysiology and the identification of potential therapeutic targets.

Utilizing a biotinylation approach, we successfully extracted and enriched surface proteins from human sensory-like neurons derived from a patient with FD and a healthy control, used as *in vitro* model systems. On the methodological level, we show that Matrigel proteins were undetectable by mass spectrometry after six weeks of incubation. This finding extends the applicability of the enrichment strategy to a wide range of cell culture systems that rely on vessel coating, including iPSC and iPSC-derived cell types. Given the limited information available on the stability of Matrigel, it is noteworthy that the related basement membrane matrix Geltrex (Thermo Fisher Scientific, Waltham, MA, USA) is reported to remain stable for up to two weeks. The prolonged incubation period of six weeks, combined with the applied washing steps, likely explains the absence of Matrigel proteins in our analysis.

On the context level, surface expression of heat-sensing transient receptor potential vanilloid 1 (*TRPV1*) was not detected in our sensory-like neurons despite confirmed mRNA expression [2]. In fact, recent studies using similar differentiation protocols reported low numbers of cells responsive to the *TRPV1* agonist capsaicin [59]. Hence, our data support the notion that mRNA expression does not reliably reflect ion channel surface expression. The same phenomenon could apply to the absence of voltage-gated sodium channel Na_v_1.8, while its functional expression in iPSC-derived sensory-like neurons has long been debated [59].

Consistent with the initial characterization of the applied differentiation strategy for sensory-like neurons, our results revealed the expression of *RET* and *NTRK2*, while *NTRK1* was notably absent [60]. This expression pattern is indicative of non-peptidergic sensory-like neurons which was indeed identified as the functionally affected neuronal subclass in the context of FD [50,51]. Notably, a recent study has questioned the traditional peptidergic versus non-peptidergic dualism in human dorsal root ganglia (DRG), proposing instead the existence of twelve distinct neuronal subtypes, offering a more granular view of sensory neuron classification [61]. However, the surface expression observed in our neurons did not fully correspond to any of the described cellular subtypes, rendering the applicability and precise classification in iPSC-derived sensory-like neurons ambiguous in our study.

Analysis of protein-protein interactions among surfaceome proteins differentially regulated in the FD and healthy control neurons revealed several clusters associated with neuronal network formation and integrity. Supporting this, previous studies have reported an increase in neuronal soma size [62] and a decrease in neurite outgrowth in DRG neurons in a FD mouse model [18]. Interestingly, one cluster contained several components of the extracellular matrix, which were captured due to their accessibility for biotin labeling. Lysosomal dysfunction, known to be impaired in FD [63], directly affects matrix secretion [64]. A recent proteomic study of plasma from FD patients and controls further suggested extracellular matrix remodeling, corroborating our findings [65]. As extracellular matrix proteins are predominantly not secreted by neurons [66], this finding could also be attributed to FD- or even cell line-related differences with regard to the few remaining non-neuronal cells that are inevitable in such iPSC-derived cell cultures.

Neuropathic pain is a hallmark in FD patients and current analgesic treatment options are insufficient [67]. We used an iPSC line derived from a patient with FD and carrying a pathogenic genetic variant in *GLA* and compared data with a healthy control cell line, both utilized as an *in vitro* model system to establish our protocol for surfaceome analysis. We succeeded in the enrichment of surface proteins and also found a distinct deregulation of surfaceome proteins when comparing data from both cell lines, however, our data can only be of descriptive nature given the proof-of-principle approach followed in our study.

With this limitation in mind, we identified distinct deregulations in the surfaceome of sensory-like neurons obtained from a FD patient versus a healthy control. We observed an upregulation of the calcium voltage-gated channel auxiliary subunit alpha2delta 3 (CACNA2D3), which has been linked to heat pain deficiency in animal models and may play a role in thermal pain responses in humans. This is an interesting finding given that heat and fever trigger pain in FD patients. We further found an upregulation of the neuronal membrane glycoprotein m6a (GPM6A) which is associated with nerve regeneration and which could be associated with peripheral denervation as a hallmark of FD. The epidermal growth factor receptor (EGFR), which was also downregulated in our analysis, has been associated with pain signaling, and its inhibition has shown pain relief in neuropathic pain patients. Collectively, these findings are examples of how surfaceome analysis of membrane proteins expressed in neurons may be utilized to investigate disease pathophysiology.

One methodological limitation of our study is that the accessibility of cytosolic proteins to/ for biotin in apoptotic cells may introduce biases in surface protein comparisons across different neuronal lines. While we attempted to minimize viability-related batch variation through multiple independent differentiations, further studies with broader patient cohorts and improved methodological controls are warranted to validate our findings. Another context-related limitation of our study lies in the reliance on a single FD variant from one patient, which does not represent the spectrum of FD presentations. Surface protein expression could vary among other variants, particularly those exhibiting residual enzyme activity, which does not allow any generalization of our findings. This is particularly true given the absence of an isogenic control line.

Our data represent the pilot analysis of surfaceome composition in human iPSC-derived sensory-like neurons. The integration of biotin-based enrichment with *in vitro* differentiation of target cell types paves the way for a novel approach to surface protein investigation in future studies involving iPSC-derived neurons.

## Supporting information

S1 Fig Exemplary image of gel lanes loaded and run with samples derived from biotin-treated (right) and -untreated (middle) cell cultures following purification in NeutrAvidin columns. (PNG)

S1 Table TaqMan Gene Expression Assays used for quantitative real-time polymerase chain reactions. (XLSX)

S2 Table Proteins identified by mass spectrometry after biotinylation-based surfaceome enrichment in FD and/or control neuron cultures and all three independent experiments. Proteins are ordered from highest (top) to lowest (bottom) intensity/abundance. The given parameters for FD versus control LFQ intensity ratio, accumulated LFQ intensity, and p-value were used in the volcano plot shown in Fig 2. Abbreviations: FD, Fabry disease; LFQ, label-free quantitation.
(XLSX)

S3 Table Results of functional annotation clustering in the top 100 most abundant proteins, performed with the Database for Annotation, Visualization and Integrated Discovery (DAVID). (XLSX)

S4 Table Proteins detected by mass spectrometry in mere Matrigel probes without seeding of sensory-like neurons. (XLSX)

S5 Table Proteins deregulated in FD sensory-like neurons compared to healthy control, ordered from highest (top) to lowest (bottom) difference. Proteins were assessed for surface expression using CSPA and UniProt databases, and considered part of the surfaceome if one of the criteria was fulfilled. Abbreviations: CSPA, cell surface protein atlas; FD, Fabry disease.
(XLSX)

S6 Table Assessment of deregulated surface protein interaction, generated with the Search Tool for the Retrieval of Interacting Genes (STRING) and underlying the network presented in Fig 4. Interaction between two proteins (nodes), respectively, was rated based on eight categories and allocated to a combined score.
(XLSX)
