# Supplementary figures and images for: Human sensory-like neuron surfaceome analysis
Source: PLoS One. 2025 Apr 2;20(4):e0320056. doi: 10.1371/journal.pone.0320056 (PMC11964241; doi:10.1371/journal.pone.0320056)

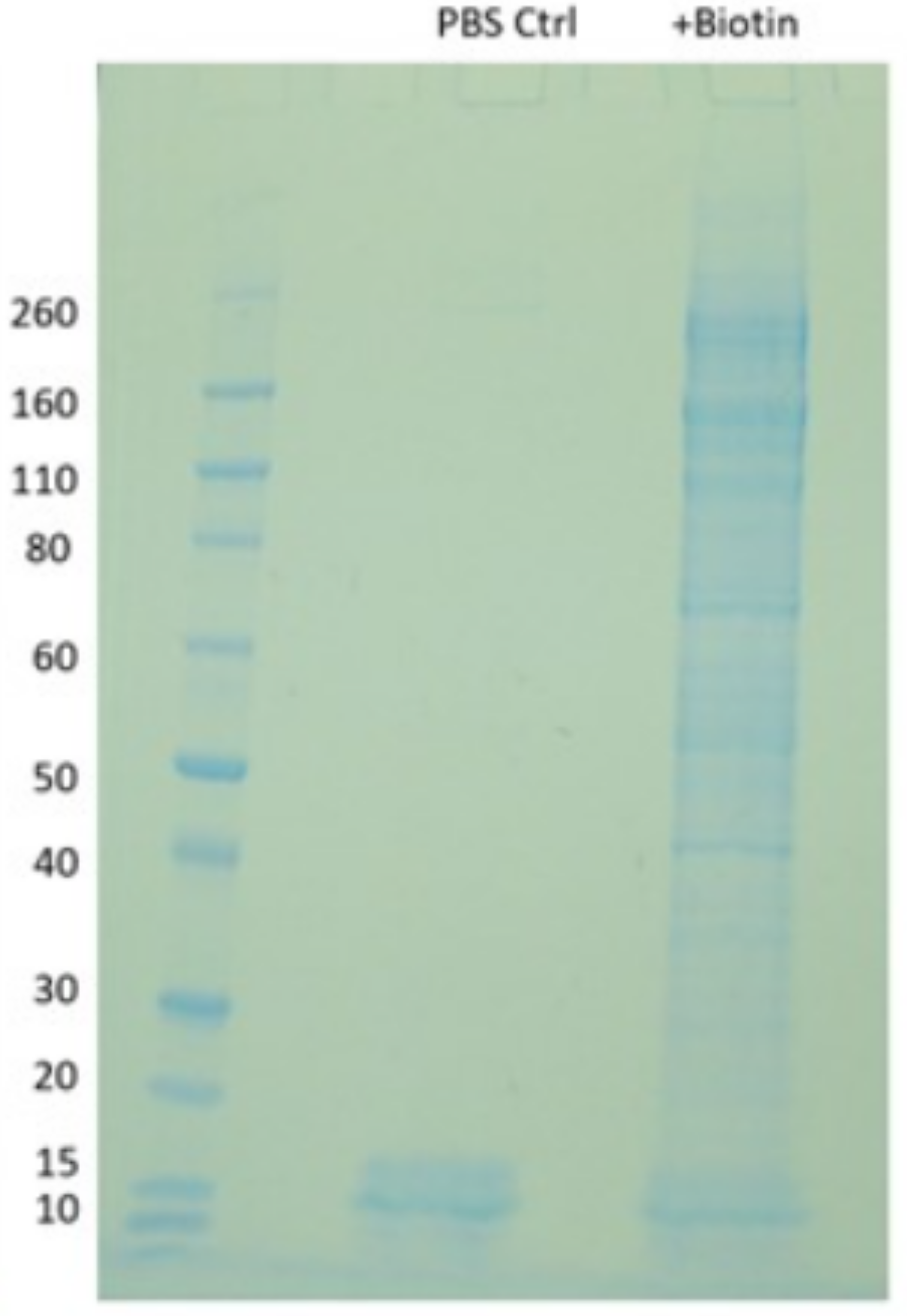

Supplement: S1 Fig — (PNG) [file pone.0320056.s001.png]
